# Mining proteomic data to expose protein modifications in *Methanosarcina mazei* strain Gö1

**DOI:** 10.3389/fmicb.2015.00149

**Published:** 2015-03-05

**Authors:** Deborah R. Leon, A. Jimmy Ytterberg, Pinmanee Boontheung, Unmi Kim, Joseph A. Loo, Robert P. Gunsalus, Rachel R. Ogorzalek Loo

**Affiliations:** ^1^Department of Chemistry and Biochemistry, University of California, Los AngelesLos Angeles, CA, USA; ^2^Microbiology, Immunology, and Molecular Genetics, University of California, Los AngelesLos Angeles, CA, USA; ^3^Department of Biological Chemistry, David Geffen School of Medicine, University of California, Los AngelesLos Angeles, CA, USA; ^4^UCLA-DOE Institute for Genomics and Proteomics, University of California, Los AngelesLos Angeles, CA, USA

**Keywords:** *S*-layers, archaeal surface proteins, *Methanosarcina mazei*, prokaryotic glycosylation, membrane proteins, concanavalin A

## Abstract

Proteomic tools identify constituents of complex mixtures, often delivering long lists of identified proteins. The high-throughput methods excel at matching tandem mass spectrometry data to spectra predicted from sequence databases. Unassigned mass spectra are ignored, but could, in principle, provide valuable information on unanticipated modifications and improve protein annotations while consuming limited quantities of material. Strategies to “mine” information from these discards are presented, along with discussion of features that, when present, provide strong support for modifications. In this study we mined LC-MS/MS datasets of proteolytically-digested concanavalin A pull down fractions from *Methanosarcina mazei* Gö1 cell lysates. Analyses identified 154 proteins. Many of the observed proteins displayed post-translationally modified forms, including *O*-formylated and methyl-esterified segments that appear biologically relevant (i.e., not artifacts of sample handling). Interesting cleavages and modifications (e.g., *S*-cyanylation and trimethylation) were observed near catalytic sites of methanogenesis enzymes. Of 31 *Methanosarcina* protein *N*-termini recovered by concanavalin A binding or from a previous study, only *M. mazei S*-layer protein MM1976 and its *M. acetivorans* C2A orthologue, MA0829, underwent signal peptide excision. Experimental results contrast with predictions from algorithms SignalP 3.0 and Exprot, which were found to over-predict the presence of signal peptides. Proteins MM0002, MM0716, MM1364, and MM1976 were found to be glycosylated, and employing chromatography tailored specifically for glycopeptides will likely reveal more. This study supplements limited, existing experimental datasets of mature archaeal *N*-termini, including presence or absence of signal peptides, translation initiation sites, and other processing. *Methanosarcina* surface and membrane proteins are richly modified.

## Introduction

Knowledge about Archaea and their proteins is limited, making their characterization important. Fortunately, tools are available to identify proteins at high throughput, while bioinformatic analyses can overlay existing knowledge onto this kingdom. Nevertheless, protein modifications unique to these organisms and/or rare in well-studied microbes may elude us, primarily because high throughput proteomic methods focus on matching peptide fragment data to what can be anticipated, primarily from the genome sequence.

Progress in understanding archaeal cell surface structures has been hindered by the limited availability of experimental results, and any new protein modifications that are revealed may hint at function. Here peptide tandem mass spectrometry (MS/MS) datasets from previous investigations of *Methanosarina S*-layer and surface-exposed proteins (Francoleon et al., 2009; Rohlin et al., 2012), as well as from mixtures recovered by concanavalin A binding were selected for further analysis, motivated by interest in how protein modifications can impact organisms' interactions with their environment and with other organisms.

The above-mentioned emphasis on matching high throughput proteomic data to predictions means that *unassigned mass spectra, (often 90% of all data) are ignored* (Savitski et al., 2005; Baumgartner et al., 2008; Falkner et al., 2008; Menschaert et al., 2009; Hahne et al., 2013). In principle, unassigned proteomic data could be a treasure trove. In practice, its value depends on sample complexity (whether it contains ~5000 or 50,000 tryptic peptides), the protein and/or peptide separation strategies employed for its acquisition [single-dimension liquid chromatography (LC) of tryptic peptides vs. two-dimensional polyacrylamide gel electrophoresis (2D-PAGE) of proteins followed by peptide LC], and on the quality of the tandem mass spectrometry (MS/MS) data.

There are many reasons why peptide tandem mass spectra may be unassigned in these complex experiments:

1. The spectra may be poor in quality and/or low in information content.
2. The MS/MS spectra may be derived from a mixture, rather than from a single peptide.
3. The spectra may be derived from a peptide that is modified in a manner not considered by the algorithm.

High throughput workflows compromise between *speed* and *depth of analysis*, with “success” generally assessed by the number of proteins identified at a specified peptide or protein false discovery rate (FDR). This gene-centric approach does not differentiate between modified and/or processed forms of proteins. Typically, only modifications related to sample handling are considered (Schmidt et al., 2005) to balance *sensitivity* (number of peptides or proteins identified) with *specificity* (low false discovery rate). If post-translational modifications (PTMs) are pursued in high-throughput environments, modified peptides are typically enriched *via* PTM-specific immunoprecipitation or other affinity capture methods; e.g., phosphopeptide binding to TiO_2_.

Skillful data mining can recover valuable information about unanticipated modifications from high throughput proteomic data. Some strategies include:

1. Performing an error-tolerant search (Creasy and Cottrell, 2002); i.e., a second search limited to proteins detected in the initial search. It attempts to match mass spectra by considering one additional modification-type from the UniMod collection or single amino acid substitutions (Creasy and Cottrell, 2004). It also relaxes enzyme specificity, e.g., only the *N*- or *C*-terminus of a peptide must conform to trypsin's known cleavage specificity, rather than both.
2. Searching against the genome sequence translated in 6-reading frames (three forward and three reverse strand translations). This approach overcomes DNA sequencing errors, missed open reading frames, incorrect start or stop sites, and alternate initiation. It can also be employed in an error-tolerant mode to overcome nucleobase substitutions.
3. Attempting to match spectra by assuming that one residue's mass has been shifted by some amount (within a specified range) or by clustering related spectra (Savitski et al., 2006; Bandeira et al., 2007; Falkner et al., 2008; Wilhelm and Jones, 2014).
4. Manual or computer-assisted *de novo* interpretation of mass spectra.

Evaluating accuracy is challenging for any data mining strategy. When searches are limited to relatively common modifications, it may be possible to calculate separate false discovery rates for the modified peptides. But when almost anything is possible, confirmatory information must be sought elsewhere.

Here, we illustrate how proteomic datasets can be mined to recover information about *Methanosarcina mazei* protein modifications and describe some characteristic mass signatures that assist in validating that modifications are present. Clearly, methods including antibody blotting, functional group specific staining, and chemical derivatization also provide essential verification. The focus of this manuscript is on mining existing data to recover information about unanticipated modifications. The knowledge may suggest protein forms (proteoforms) to track in future studies, follow-up experiments to confirm the modifications, it may hint at protein function, or it may simply improve protein annotations.

## Materials and methods

### Cell cultivation

*M. mazei* Gö1 was grown at 37°C as single cells (non-aggregated) in *pH* 6.8 basal mineral medium prepared by the Hungate technique and supplemented with 0.05 M methanol as the sole source of carbon and energy (Sowers et al., 1993). Medium osmolarity was defined by 0.2 M NaCl. Cultivation employed 10-mL anaerobic tubes (Difco, Sparks, MD) sealed with a N_2_-CO_2_ (4:1) atmosphere. Cultures were harvested at an average OD_600_ of ~1.5.

### Crude extract/lysate preparation

Eight tubes of 10-mL anaerobic cultures were unsealed and their contents were transferred to 15-mL Falcon™ centrifuge tubes. Cells were sedimented at room temperature for 10 min in a swing bucket rotor at 1125 × *g*. After centrifugation, 500 μL of chilled lysis buffer was added to each tube [2% (*w/v*) CHAPS (3-(3-cholamidopropyl) dimethylammonio-1-propanesulfonate) in *pH* 7.5, 50 mM Tris, 0.15 M NaCl, 1 mM CaCl_2_, and 1 mM MnCl_2_ supplemented with 2.25 μL Sigma P8465 protease inhibitor]. The cell pellets were disrupted further by multiple freeze/thaw cycles interspersed with vortexing. The lysates were transferred to 1.5 mL microcentrifuge tubes for centrifugation at 16,000 × *g* for 15 min at 4°C. The soluble lysate was retained for analysis.

### Concanavalin a glycoprotein enrichment

Our previous purification was modified slightly (Francoleon et al., 2009). Briefly, each of four 5-mL centrifuge columns (Pierce, Rockford, IL) was loaded with a 2 mL slurry of Con A-coupled agarose beads (Vector Laboratories, Burlingame, CA). The beads were washed with 3 mL of 50 mM Tris, 0.15 M NaCl (*pH* 7.5) seven times, followed by six equilibrating washes with 2 mL binding buffer [BB, 50 mM Tris, 0.15 M NaCl, 1 mM CaCl_2_, 1 mM MnCl_2_ (*pH* 7.5)]. After equilibration, 1 mL of lysate and 1 mL of BB buffer were added to each column for incubation in a room temperature rotor. After 30 min, the column flow through was collected by centrifugation and discarded. The protein-bound beads were washed 10 times to minimize non-specific binding: (1) five washes, each with 2 mL of BB buffer supplemented with 0.1 % Tween-20, followed by (2) five 2-mL washes with 50 mM of (NH_4_)HCO_3_ (*pH* 7.8). Glycoproteins were eluted from the lectin media twice: (1) Con A beads were incubated in 2 mL of elution buffer [50 mM (NH_4_)HCO_3_/0.2 M methyl-α-D-mannopyranoside/0.2 M methyl-α-D-glucopyranoside (*pH* 7.8)] for 10 min at room temperature, and centrifuged to recover the eluate. (2) The elution was repeated with 1 mL of buffer. The combined eluate was concentrated to 1.6 μg/μL (Pierce BCA assay) by ultrafiltration through an Amicon® 50 kDa cut-off cellulose membrane (Millipore, Billerica, MA).

Multiple enrichments were also performed on a smaller scale in a manner similar to that described above, but without ultrafiltration.

### In-solution trypsin, Glu-C and Asp-N proteolysis

Each proteolytic digestion used 50 μL of concentrated Con A eluate (~78 μg of total protein) which, prior to digestion, was precipitated at −20°C overnight in 9 volumes of chilled acetone. Protein precipitate was recovered by centrifugation at 4°C, 16,000 × *g* for 20 min. Pellets were washed in 500 μL of chilled 80% acetone/10% methanol/0.2% acetic acid.

For trypsin digestion, the protein was resuspended in 20 μL of room temperature dimethyl sulfoxide (DMSO) with 600 rpm shaking for 30 min (Ytterberg et al., 2006). The solution was diluted to 30% DMSO/50 mM (NH_4_)HCO_3_ and sequencing grade trypsin (Promega, Madison, WI) was added at a 1:20 enzyme:protein ratio (*w/w*). Digestion proceeded overnight at 37°C with 300 rpm shaking.

For Glu-C digestion, precipitated protein was resuspended in 131 μL of *pH* 7.8, 25 mM (NH_4_)HCO_3_and 1:20 (*w/w*) Glu-C:protein (sequencing grade *Staphylococcus aureus* Protease V-8, Roche, Indianapolis, IN). The reaction proceeded for 4 h with 300 rpm shaking in a 25°C incubator.

Asp-N cleavage employed sequencing grade *Pseudomonas fragi* Asp-N (Roche) at 1:20 Asp-N:protein (*w/w*). The protein pellet, resuspended in 131 μL of *pH* 7.5, 25 mM Tris buffer, was incubated for 4 h at 37°C with 300 rpm shaking.

#### Nano-HPLC and data dependent MS/MS

Aliquots (~5 μg) of trypsin- and Glu-C-digested peptides were dried by vacuum centrifugation and resuspended in 125 μL of 5% formic acid (FA) to ~40 ng/μL. Asp-N digested proteins (~10 μg) were desalted using a C-18 spin column (Pierce), dried, and resuspended to ~40 ng/μL in 5% formic acid.

Peptide mixtures were analyzed by liquid chromatography-tandem mass spectrometry (LC-MS/MS) with ESI (electrospray ionization) on an Applied BioSystems QSTAR® Pulsar XL quadrupole time-of-flight mass spectrometer equipped with a nanoelectrospray interface (Protana), a Proxeon (Odense) nano-bore stainless steel emitter (30 μm ID), and an LC Packings nano-LC system as described previously (Francoleon et al., 2009). Homemade pre- (150 × 5 mm) and analytical (75 μm × 150 mm) columns were packed with Jupiter Proteo C12 4-μm resin (Phenomenex). Typically 6 μL of sample was loaded onto the precolumn, washed with 0.1% FA for 4 min and transferred to the LC column. The 200 nL/min mobile phase gradient employed 3–6% B in 6 s, 6–24% B in 18 min, 24–36% B in 6 min, 36–80% B in 2 min, and 80% B for 7.9 min. The column was equilibrated with 3% B for 15 min prior to the next run. Eluents used were 0.1% FA (aq) (solvent A) and 95% CH_3_CN containing 0.1% FA (solvent B).

Peptide product ion spectra were recorded automatically by IDA (information-dependent analysis) software on the mass spectrometer. Protein sequence searches employed a conservative mass tolerance of 0.3 Da for both precursor and product ions, and 1 (trypsin) or 2 (Asp-N and Glu-C) missed cleavages. Proteins hits were accepted based on ≥2 ascribed peptides, at least of one which possessed a MOWSE score ≥26 (*p* ≤ 0.02) with MuDPIT scoring. Identifications based on single peptides are presented separately. Correspondences between MS/MS spectra and all ascribed sequences were also verified manually.

#### Nano-HPLC and data independent acquisition MS/MS

The peptide mixtures described above were also analyzed by LC-ESI-MS/MS on a Xevo™ quadrupole time-of-flight MS (Waters Corporation) equipped with a Universal NanoFlow Sprayer interface and pre-cut Pico Tip Emitter (360 μm OD × 20 μm ID, 10 μm tip; 2.5″ long), connected on-line to a nanoACQUITY® UltraPerformance® HPLC system (Waters Corporation). The nanoACQUITY® system was equipped with Waters' 5 μm Symmetry C_18_, 180 μm × 20 mm reversed-phase trap and 1.7 μm BEH130 C_18_, 75 μm × 100 mm reversed-phase analytical columns. Both columns were maintained at 40°C. Typically 3 μL of samples were injected onto the precolumn in aqueous 1% CH_3_CN/0.1% FA at a flow rate of 5 μL/min for 3 min. Mobile phase A was water with 0.1% FA (*v/v*) and mobile phase B was CH_3_CN with 0.1% FA. After desalting and concentrating in the trap column, peptides were transferred to the analytical column and resolved by a gradient of 3–60% mobile phase B delivered over 30 min at a flow rate of 300 nL/min, followed by a 15 min wash with 95% B and 15 min re-equilibration at the initial conditions (3% B, 97% A).

The Xevo™ quadrupole time-of-flight MS was operated in positive ion, V-mode with an average resolution of 9500 FWHM. Full scan mass spectra were acquired from 50 to 2000 *m/z*. LC-MS and LC-MS^E^ data were collected in alternating low and high collision energy modes throughout the run (Silva et al., 2005), with each spectrum acquired for 1 s per mode.

Proteins were identified using the ProteinLynx Global SERVER™ version 2.4 search engine (PLGS, Waters Corporation). All ions were lock mass corrected, de-isotoped, and decombulated (charge state reduced). PLGS software ascribed collision induced dissociation (CID) product ions to their precursor peptides by time-aligning low- and high-energy-detected ions with a retention time tolerance of approximately ±0.05 min. Sequence searches were restricted to fully tryptic products with up to one missed cleavage, variable methionine oxidation and *N*-terminal Gln/Glu conversion to pyro-Glu, and peptide and product ion tolerances of 10 and 25 ppm, respectively. Proteins hits were accepted based on ≥2 ascribed peptides, each with 3 or more product ions, and at least seven fragment ions per protein. Correspondences between MS/MS spectra and ascribed sequences were also evaluated manually.

## Results and discussion

We identified 154 proteins from concanavalin A pull down fractions and cell surface labeling. The following sections describe these proteins and the additional information that can be recovered by data mining.

### Identificationl of concanavalin a interacting proteins

The archaeal cytoplasmic membrane has been described as fulfilling the role of the eukaryotic endoplasmic reticulum (Yurist-Doutsch et al., 2008) because archaeal glycosylation machinery is membrane-bound. Cell *N*-linked glycoproteins are expected to localize to the cytoplasmic membrane or the associated outer cell envelope region (Eichler, 2003; Albers et al., 2006; Messner, 2009). Thus, glycoprotein capture methods (e.g., lectin affinity chromatography) complement cell surface labeling (Francoleon et al., 2009) for enriching archaeal surface and membrane proteins.

To support ongoing studies characterizing protein glycosylation, *M. mazei* cell lysate proteins were lectin affinity-captured through direct and indirect binding using Con A, for which the subset of direct binders typically includes glycoproteins containing α-*D*-mannose and α-*D*-glucose (Kornfeld and Ferris, 1975; Baenziger and Fiete, 1979; Debray et al., 1981; Jaipuri et al., 2008). Here, we describe what can be learned from LC-MS/MS analyses of the Con A eluate with subsequent data mining. Further studies that employed additional dimensions of separation [e.g., hydrophilic interaction chromatography (HILIC) followed by reversed phase liquid chromatography], in concert with advanced ion activation techniques including infrared multiphoton dissociation (Zubarev, 2004; Cooper et al., 2005) will be discussed elsewhere (Leon et al., in preparation). Those methods greatly increase the numbers of glycoproteins observed, improve ability to localize glycosylation sites, and characterize individual glycan chains. Nevertheless, useful information can be gleaned from these initial LC-MS/MS experiments.

Not all proteins recovered from the Con A eluate are necessarily glycosylated. Indirect binding partners do not bind Con A directly, but associate with one or more direct (glycosylated) interactors; e.g., other subunits of a non-covalent complex. From *M. mazei* Gö1, 99 Con A eluate proteins were identified by LC-MS/MS with ≥2 peptides (Table 1). An additional 55 protein identifications based on a single peptide are presented in Supplemental Table S-1.

**Table 1:** **proteins detected by two or more peptides from concanavalin A eluate**.

**Uniprot accession**	**MM#**	**Name**	**Unique peptides observed**	**Mascot protein score**	**Exprot^a^**	**SignalP^b^**	**SecP^c^**	**LipoP^d^**
Q8Q0X9	MM0001	Dipeptide ABC transporter	5	229	1		Y	
Q8Q0X8	MM0002	Dipeptide ABC transporter	20	555	1		Y	
Q8Q0S0	MM0066	Hypothetical protein	5	216	2	Y	Y	
Q8Q0K8	MM0128	Lon protease, membrane-bound	8	276				
Q8Q0F2	MM0184	Rps3Ae	2	51				
Q8Q035	MM0303	Hypothetical protein	2	39	1		Y	
Q8PZM5	MM0467	*S*-layer paralog	17	606		Y	Y	
Q8PZ66	MM0628	Methylenetetrahydromethanopterin reductase	6	276				
Q8PZ61	MM0633	Multiheme cytochrome *c*, hypothetical	2	90			Y	
Q8PZ47	MM0647	Oligosaccharyl transferase	2	91			Y	
Q8PYZ8	MM0700	HppA	3	39		Y^*^	Y	
Q8PYY5	MM0714	Fructose bis phosphate aldolase	2	29				
Q8PYY3	MM0716	Hypothetical protein	3	105	1	Y	Y	
Q60187	MM0779	AtpB	6	189				
Q60186	MM0780	AtpA	11	368				
Q60184	MM0782	AtpC	4	154				
Q60183	MM0783	AtpE	4	34,13				
F1SVJ4	MM0784	AtpK	5	365	1	Y^*^	Y	
O59659	MM0785	AtpI	4	115			Y	
Q8PYS4	MM0786	AtpH	2	75				
Q8PYJ5	MM0866	Periplasmic serine protease	4	242	1		Y	
Q8PYIO	MM0882	Hypothetical protein	3	106			Y	
Q8PYE0	MM0922	Eif5a, IF5A	2	131				
Q8PY73	MM0991	Thioredoxin	5	150	2	Y		Y
Q8PY52	MM1012	Rpl1P	3	62				
Q8PY39	MM1025	ThiC	3	58				
Q8PXZ6	MM1070	MtaA1 methylcobalamin:CoM methyltransferase	7	374				
Q8PXZ5	MM1071	4Fe:4S ferredoxin, hypothetical	2	121				
Q8PXZ3	MM1073	MtaC2 methyl corrinoid protein	6	230				
Q8PXZ2	MM1074	MtaB2	9	250				
Q8PXZ1	MM1075	Putative regulatory protein	2	92	1		Y	
Q8PXX0	MM1096	Thermosome, gamma subunit	5	137				
Q8PXW0	MM1106	Putative phosphoserine phosphatase	5	81				
Q8PXV8	MM1108	Mtd, F420-dependent methylenetetrahydromethanopterin dehydrogenase	3	126				
Q8PXI2	MM1236	HtpX protease	2	25		Y	Y	
Q8PXH8	MM1240	Methyl CoM reductase, alpha subunit	7	237				
Q8PXH7	MM1241	Methyl CoM reductase, gamma subunit	10	563				
Q8PXH4	MM1244	Methyl CoM reductase, beta subunit	13	467				
Q8PXG8	MM1250	Cation transporter	2	66		Y^*^	Y	
Q8PXG7	MM1251	Cation transporter	2	109		Y^*^	Y	
Q8PX89	MM1333	Zinc ABC transporter	10	486	2	Y		Y
Q8PX82	MM1340	Pyruvate synthase, alpha subunit	2	61				
Q8PX60	MM1362	Putative aliphatic sulfonate binding	11	556	2	Y	Y	
Q8PX58	MM1364	*S*-layer paralog	28	1093	1	Y	Y	
Q8PX43	MM1379	Thermosome, alpha subunit	10	329				
Q8PWZ8	MM1424	SecF	2	85	1	Y	Y	
Q8PWZ7	MM1425	SedD	3	144		Y	Y	
Q8PWX0	MM1456	USP-like	4	153				
P80650	MM1540	MtrH	9	276				
P80656	MM1541	MtrG	4	192			Y	
P80654	MM1542	MtrF	2	105			Y	
O59640	MM1543	MtrA	6	309				
P80655	MM1544	MtrB	4	222			Y	
O59638	MM1545	MtrC	5	127			Y	
P80653	MM1546	MtrD	2	133		Y	Y	
P80651	MM1547	MtrE	4	117		Y	Y	
Q8PWN5	MM1549	Na/proline symporter	4	211		Y^*^	Y	
Q8PWN3	MM1551	Hypothetical protein	4	75	1	Y	Y	
Q8PWM3	MM1561	ABC transporter, tungsten binding protein	2	72		Y^*^	Y	
Q8PWJ6	MM1589	Surface layer protein B	3	128	1	Y	Y	
Q8PWE2	MM1647	MtaB1	14	357				
Q8PWE1	MM1648	MtaC1	10	533				
Q8PWA1	MM1695	Hypothetical protein	10	425	2	Y	Y	Y
Q8PW83	MM1713	Hypothetical protein	2	173			Y	
Q8PW41	MM1760	Rps2p 30S ribosomal protein S2	3	104			Y	
Q8PW15	MM1789	Hypothetical protein	2	64			Y	
Q8PVY9	MM1816	Conserved protein	6	238	1	Y	Y	
Q8PVW4	MM1843	HdrE, heterodisulfide reductase	6	210		Y	Y	
Q8PVV1	MM1859	DdpA, ABC transporter	3	29	1	Y	Y	
Q8PVM4	MM1939	Glutamine binding protein	2	115	2	Y		Y
Q8PVI7	MM1976	*S*-layer protein, (identified)	151	5310	1	Y	Y	
Q8PVI6	MM1977	Hypothetical protein	3	118	2	Y	Y	Y
Q8PVG6	MM1999	Hypothetical protein	4	103	2	Y	Y	Y
Q8PVG5	MM2000	Hypothetical protein	4	82	2	Y	Y	Y
Q8PVD2	MM2033	Stomatin-like protein	2	52		Y^*^		
Q8PVA2	MM2069	Iron III dicitrate binding protein	5	179	2	Y	Y	Y
Q8PV27	MM2147	SecY	2	67			Y	
Q8PV17	MM2157	Rpsllp, 30S ribosomal protein S11p	2	81			Y	
Q50227	MM2171	Cytochrome *b*, F420 non-reducing hydrogenase II	2	87			Y	
Q8PUU5	MM2234	Hypothetical protein	2	94	1	Y	Y	
Q8PUR8/AAM31960	MM2264	Ef1A	10	301				
Q8PUM8	MM2305	Na/proline symporter	2	51		Y	Y	
Q8PUL4	MM2320	EchA	3	75		Y^*^	Y	
Q8PU81	MM2460	Dipeptide oligopeptide binding protein	16	556	2	Y	Y	Y
F1SVE1	MM2479	FpoO, F420H2 dehydrogenase	2	83				
F1SVH9	MM2481	FpoM, F420H2 dehydrogenase	2	63		Y	Y	
F1SVK0	MM2482	FpoL, F420H2 dehydrogenase	2	91		Y	Y	
F1SVE0	MM2483	FpoK, F420H2 dehydrogenase	2	64		Y	Y	
Q8PU59	MM2487	FpoH, F420H2 dehydrogenase	3	97		Y^*^	Y	
P27094	MM2505	DnaK, chaperone protein	13	451				
Q8PU26	MM2531	Hypothetical protein	3	113	1		Y	
Q8PTZ0	MM2567	ABC transporter, periplasmic binding protein	14	420	2	Y		
Q8PTY1	MM2576	Ferrous iron transport protein B	8	276			Y	
Q8PTS5	MM2637	Ion channel transporter	3	58			Y	
Q8PTQ7	MM2656	Peptidyl-prolyl cis-trans isomerase	2	28				
Q8PTI7	MM2728	Hypothetical protein	2	61	1	Y^*^	Y	
Q8PT25	MM2893	Hypothetical protein	6	193	1	Y	Y	
Q8PSQ2	MM3024	Hypothetical protein	3	65	1	Y	Y	
Q8PS84	MM3198	Lipoprotein, hypothetical	5	134				

a *Exprot (Saleh et al., 2010); 1, Predicted type I signal peptidase substrate; 2, Predicted type II signal peptidase substrate*.

b *SignalP (Bendtsen et al., 2004b); Y, Predicted signal peptide by SignalP 3.0*.

c *SecP (Bendtsen et al., 2004a, 2005); Y, Predicted substrate for leaderless secretion by SecretomeP 2.0*.

d *LipoP (Juncker et al., 2003); Y, Predicted lipoprotein*.

* *Predicted as secreted only by the SignalP eukaryotic predictor*.

Standard proteomic search strategies do not reveal unknown glycopeptides, because it is not possible to predict the amount by which peptide masses are incremented. However, manual MS/MS analysis of, e.g., HILIC fractions, reveals some precursor masses (peptides) that are obviously glycosylated, because they dissociate to release low mass-to-charge ratio (*m/z*) ions, known as oxonium ions, that are characteristic of different sugars (Mechref, 2012). Ions at 163.06 and 127.06 *m/z*; e.g., signal that hexoses are present, while 204.09, 186.09, and 168.09 *m/z* reflect present *N*-acetylhexosamines.

*Bona fide* glycosylated proteins, revealed by their oxonium ions in peptide MS/MS spectra, were identified from Con A eluate as MM0002, MM0716, MM1364, and *S*-layer protein MM1976. It is unlikely that these are the only glycoproteins within *M. mazei*. Indeed, glycoprotein-specific staining (Francoleon et al., 2009) highlights many bands. Supplemental Table S-3 lists, from the proteins observed, those first predicted by SignalP 3.0 to be secreted and then by the NetNGlyc server (Blom et al., 2004) to be potentially glycosylated. The table lists 41 candidate glycoproteins. Interestingly, protein MM0002 was not predicted by SignalP to contain a signal peptide, although it is a candidate for leaderless secretion by SecretomeP, and NetNGlyc does suggest 2 Asn sites as potentially glycosylated. Hence, the list of potentially *N*-glycosylated proteins can be longer, should proteins secreted leaderlessly also be considered. The predictions from SignalP version 4.0 comprise a subset of the version 3.0 predictions. All but two of the proteins absent from the newer algorithm's list of signal peptide-containing sequences (MM1329 and MM2033) are also candidates for leaderless secretion (according to the SecretomeP algorithm), and thus potentially *N*-glycosylated. Additional glycoproteins likely await discovery.

It should also be clarified that the tandem MS conditions and protein quantities required for high-throughput peptide identification differ from those employed in oligosaccharide and glycoprotein analysis. Glycoprotein analyses will employ 10–100 fold more material and the MS/MS conditions will be customized for each analyte.

The utility of data mining is that, by employing a relatively simple enrichment method without exhaustive chromatographic purifications, these four proteins (MM0002, MM0716, MM1364, and MM1976) were revealed as glycosylated and bearing at least hexose and N-acetylhexosamine saccharides. It also indicates how glycopeptide knowledge can be extracted from tandem mass spectrometry data, even without upstream enrichment, bonus knowledge when experiments are not specifically targeting glycosylation. Adding chromatographic dimensions will increase the number of glycopeptides detected, because glycopeptide intensities are often suppressed by co-eluting non-glycopeptides, and because the chromatographic conditions that best resolve different glycopeptides differ from those best resolving peptides, in general. Analyses probing more *M. mazei* glycopeptides and at greater depth are underway.

### Prediction of archaeal protein *N*-termini

Signal peptides are key participants in protein translocation, but our ability to predict them confidently, especially for archaeal species, has been limited by availability of experimental data (Armengaud, 2009). Even among bacteria, e.g., *Mycobacterium smegmatis*, predictions were found to be erroneous for up to 19% of protein *N*-termini (Gallien et al., 2009). Complications delineating open reading frames (ORFs) arise when multiple AUG codons lie near the DNA 5' end (ambiguity that often prompts annotators to select the longest open reading frame) (Prats et al., 1992; Kozak, 1997; Meinnel and Giglione, 2008; Fournier et al., 2012), when organisms employ alternative initiation codons; e.g., GUG or UUG (Klunker et al., 2003; Falb et al., 2006; Yamazaki et al., 2006; Meinnel and Giglione, 2008; Running and Reilly, 2009; Elzanowski and Ostell, 2013), or when translation initiates at multiple sites (Prats et al., 1992). Modifications to *N*-termini are rarely predictable, and their prevalence varies with organism. Strikingly, 14–19% of protein *N*-termini in halophiles *Halobacterium salinarum* and *Natronomonas pharaonis* are acetylated (Falb et al., 2006). Clearly, experimentally characterizing the *N*-termini of microbial proteins is important.

In a computational approach, SignalP 3.0 (Bendtsen et al., 2004b), LipoP (Juncker et al., 2003), and SecretomeP 2.0 (Bendtsen et al., 2004a, 2005) algorithms were employed to predict secreted proteins, while Exprot predictions for *M. mazei* Gö1 were obtained from Supplemental information in Saleh et al. (2010). SignalP used gram-positive, gram-negative, and eukaryotic-type models, while SecretomeP was applied employing bacterial models (Bendtsen et al., 2004a, 2005). Predictions for proteins recovered by Con A binding are displayed in Supplemental Table S-2. Predicted and experimental results are compared in the following section and indicate a tendency of prediction algorithms to over-predict signal peptide excision.

### Experimentally detected *N*- and *C*-termini

Clearly, experimental approaches tailored to recovering as many *N*-terminal peptides as possible (Ogorzalek Loo et al., 2002; Gevaert et al., 2003; Dormeyer et al., 2007; Shen et al., 2007; Russo et al., 2008; Yamaguchi et al., 2008; Gallien et al., 2009; Xu and Jaffrey, 2010; Fournier et al., 2012; Kim et al., 2013; Venne et al., 2013) provide large datasets for evaluating and enhancing prediction algorithms and improving protein database annotations. Nevertheless, datasets acquired by other experimental approaches with different goals can be harvested to yield equivalent information for a smaller number of proteins, some of which are missed by large-scale “terminalomics” studies, especially because many large-scale approaches recover only free amino termini. Data harvests are also more likely to reveal instances where multiple *N*-terminal forms are present (e.g., modified and unmodified).

Information about protein *N*-termini is not *automatically* returned by database searching algorithms. Although most algorithms now consider both excised and retained initiator methionines when attempting to match MS/MS spectra, *N*-terminal acetylation is only considered if specified in the search parameters. Because each variable modification (i.e., one which may be present *or* absent) that must be considered in the search process adds to the time required for completion and often reduces specificity, only abundant variable modifications are usually considered by high-throughput proteomics studies.

From our Con A eluate studies, some LC-MS/MS spectra spanned *M. mazei* protein *N*- or *C*-termini, allowing us to compile that information in Table 2. *M. acetivorans* C2A and *M. mazei* Gö1 *N*- and *C*-termini information obtained previously (Francoleon et al., 2009), are also included. Information was recovered by semi-tryptic and error-tolerant searches. Semi-tryptic searches seek matches for MS/MS spectra to peptides in which only one terminus matches trypsin's known cleavage specificity. Examples may include (i) peptides with non-Lys or Arg *C*-termini, but with *N*-termini reflecting cleavage after Lys or Arg, or (ii) peptide *N*-termini inconsistent with cleavage after Lys or Arg, but with *C*-terminal Lys or Arg. Error-tolerant searches, described in the Introduction, consider a large range of potential modifications.

**Table 2:**
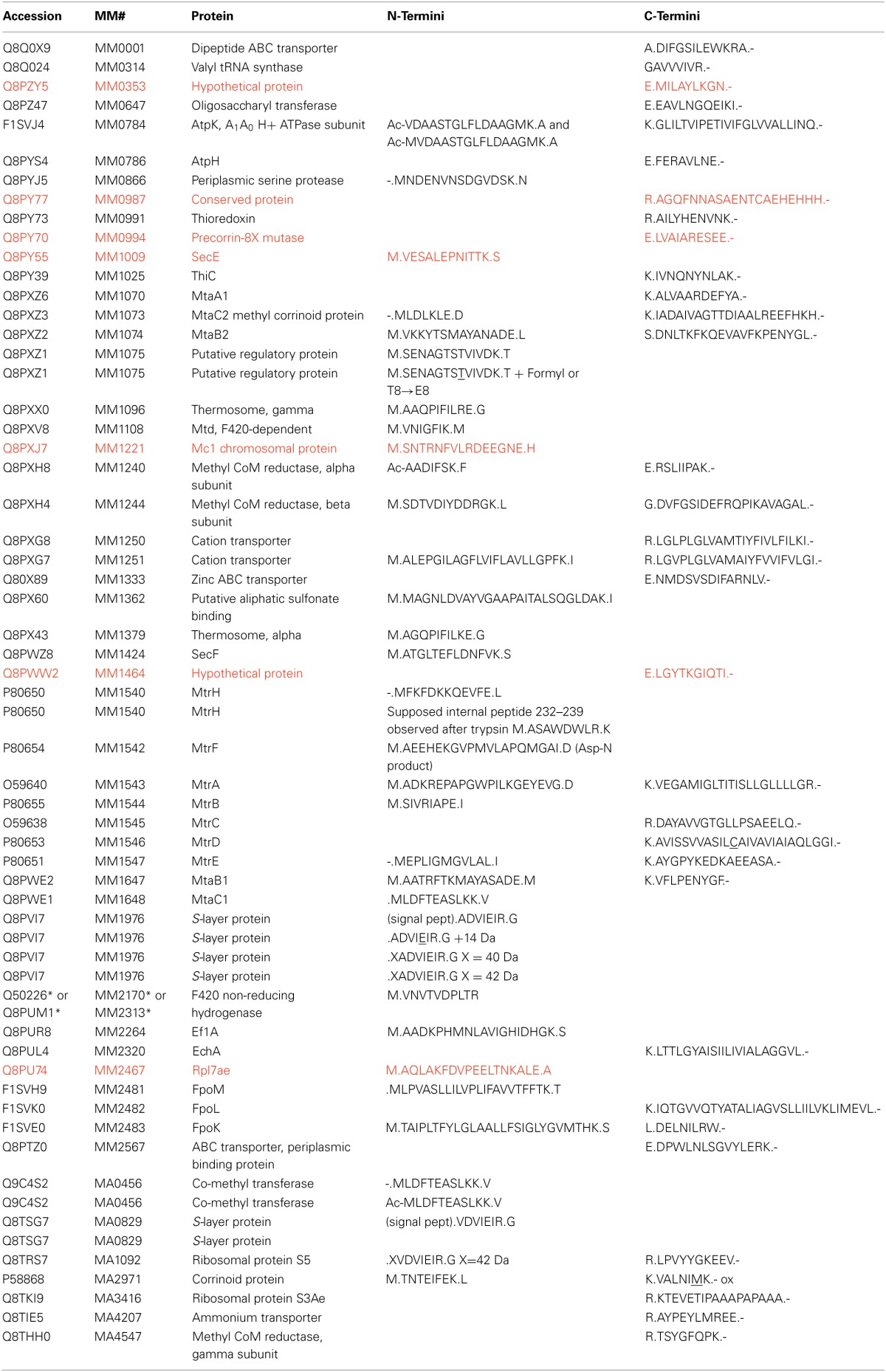
***M. mazei* and *M. acetivorans* protein termini recovered**.

^*^Several LC-MS/MS experiments were unable to differentiate between the two proteins; none of the 6 peptides identified was unique.

*Red entries correspond to identifications based on a single peptide*.

Of *M. mazei* protein *N*-termini recovered by concanavalin A binding, only *S*-layer protein MM1976 underwent signal peptide excision. These experimental results contrasted with those from prediction algorithms SignalP 3.0 (Bendtsen et al., 2004b) and Exprot (Saleh et al., 2010), which were found to over-predict the presence of signal peptides. Although SignalP 3.0 correctly predicted the MM1976 signal peptide, it also predicted leaders for 7 other proteins, while Exprot predicted signal peptides for 6 other proteins (Supplemental Table S-2). The newer algorithm SignalP 4.0 predicted leaders for only MM1362 and MM1547, in addition to MM1976. The poor correlation between prediction and experiment underscores previous conclusions about major problems predicting proteins lacking signal peptides (Antelmann et al., 2001).

Interestingly, SecretomeP 2.0, a machine-learning approach developed to predict non-classically secreted proteins in mammals and bacteria; i.e., proteins exported without a classical *N*-terminal signal peptide, predicted leaderless secretion of 14 proteins in Supplemental Table S-2 (Bendtsen et al., 2004a). Overlap for 8 of these SecretomeP 2.0 predictions with those by SignalP 3.0 is reasonable, because SecretomeP was trained on datasets of secreted protein sequences that had their signal peptides deleted. Detection of the other six proteins (MM0866, MM1009, MM1075, MM1221, MM1542, and MM1362) by our study would seem to verify these SecretomeP predictions.

*M. mazei N*-terminal peptides were recovered from 28 proteins over the course of this and previous studies. These *protein* identifications were supported by MS/MS spectra from multiple peptides in all but 3 cases. (Observing multiple peptides from a protein is one criterion for assessing confidence in the protein's identification.) Table 2 also includes data for 3 *M. acetivorans N*-termini. Heterogeneous *N*-termini were observed from 4 of the 31 proteins; e.g., Acetyl-VDAASTGLFLDAAGMK and Acetyl-MVDAASTGLFLDAAGMK were observed from A_1_A_0_ H^+^ ATPase subunit K (MM0784), indicating partial methionine excision prior to acetylation. MM0784 matched predictions in all other respects; the 4 tryptic peptides observed verified 100% of the 8-kDa proteolipid's sequence. Strong *b*_1_ ions (i.e., peptide fragments corresponding to protonated Ac-Val - H_2_O or protonated Ac-Met – H_2_O (*m/z* 142.09 and 174.06, respectively) in the MS/MS spectra confirmed the modification as *N*-acetylation (Yalcin et al., 1995). Because *b*_1_ ions are generally observed in MS/MS spectra of *N*-terminally acetylated peptides, but otherwise relatively rare, their presence provides powerful validation of the modified peptide that is independent of the statistical score; i.e., search algorithms do not accord special significance to *b*_1_ ions.

Partial methionine processing has been noted in other archaeal species (Falb et al., 2006). That the *N*-terminus of the A_1_ ATPase proteolipid subunit was found to be blocked was not surprising; blocked *N*-termini have frequently been observed for F_0_ proteolipids; e.g., *N*-formylmethionine in bacteria (*E. coli* and *Bacillus*, UniProt Accessions P68699 and P00845, respectively), yeast mitochondria (Sebald et al., 1979), and wheat (Howe et al., 1982) and spinach (P69447) chloroplasts.

**Figure 1:**
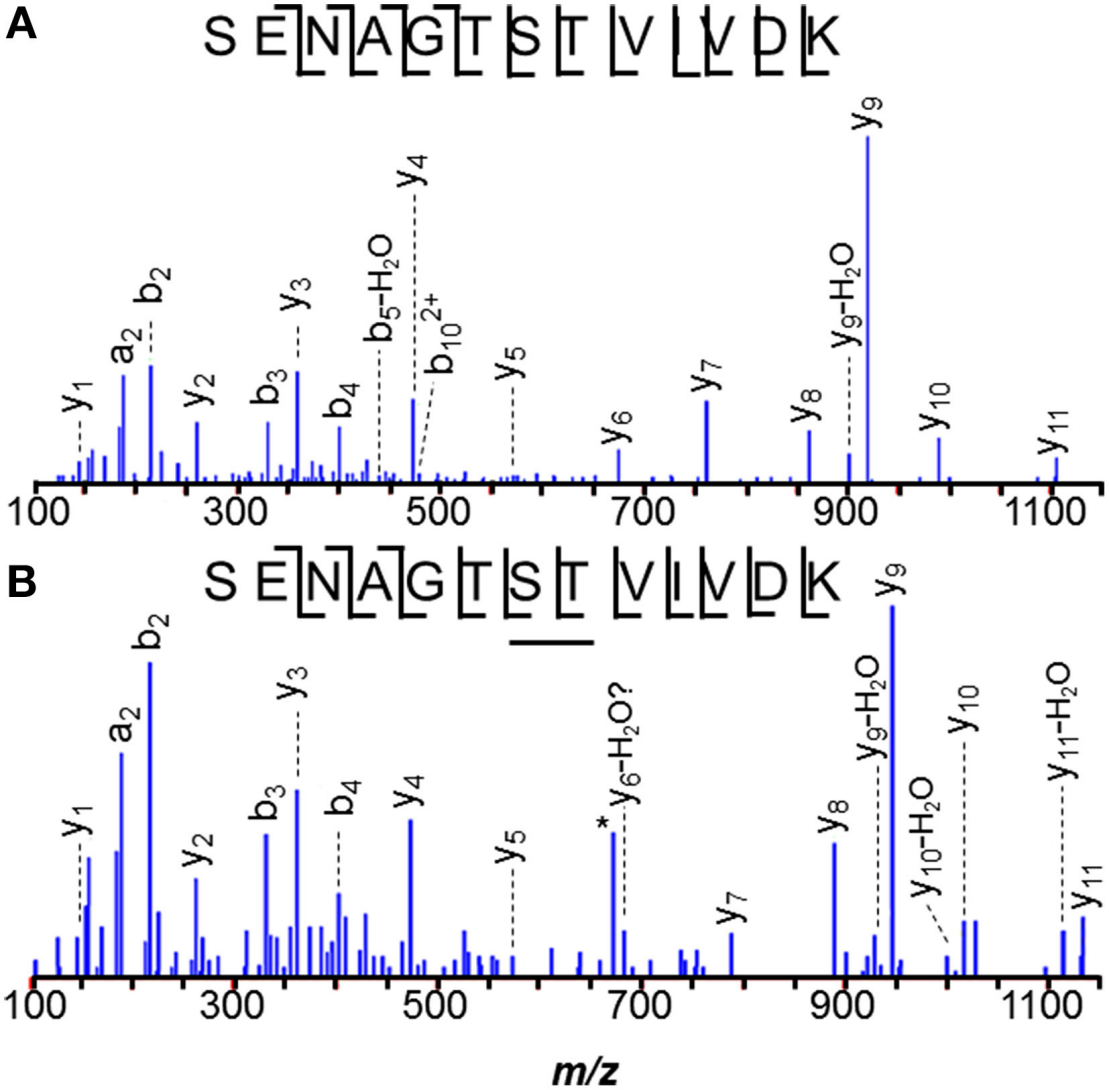
***N*-terminal peptide of *M. mazei* MM1075 (MtaR). (A)** unmodified, and **(B)** *O*-formylated at Ser_7_ or Thr_8_. Unidentified *m/z* 673, ^*^, lies 1 Da below the predicted *m/z* for unmodified y_6_.

Putative regulatory protein MM1075 (MtaR) was detected as M.SENAGTSTVIVDK (where M.S denotes methionine excision) and a formylated version, M.SENAGTSTVIVDK, modified at *S*7 or *T*8. MS/MS spectra revealed ions *y*_1_−*y*_5_, and *y*_7_−*y*_11_, localizing the modification to *S*7 or *T*8. (See Figure 1). Technically, *T*→*E*, or *S*→*D* substitutions or C_2_H_4_ addition would also match the incremented mass, but the multiple base substitutions required to convert a Thr (ACC codon) to Glu (GAA/GAG) or a Ser (UCU) to Asp (GAU/GAC) are not easily reconciled, leading us to favor interpretation as formylation. High mass accuracy measurements can distinguish addition of CO (formylation) from C_2_H_4_ (27.995 vs. 28.031 Da).

*O*-formylation may be important to controlling MM1075's function, adapting cells to acetate- and methanol-dependent growth. It's mRNA levels are 200–500 times higher in methanol- vs. trimethylamine-grown cells, and still higher for acetate culture (Hovey et al., 2005; Krätzer et al., 2009), while the operon's other genes, MM1073 and MM1074, are among the most highly regulated genes known in the Archaea (Bose et al., 2006). It will be interesting to monitor modifications of MM1075 as culture conditions are varied. For example, evidence that the ratio of formylated:unformylated MM1075 varies with; e.g., substrate or with length of time since the substrate was switched, would support a role in adaptation for the modification.

### Surface layer protein modifications

The *M. mazei* sheath or *S*-layer protein, MM1976, is one of the most abundant proteins made by the cell (Francoleon et al., 2009; Rohlin et al., 2012). Con A binding enriched cell lysates for this protein, permitting characterization of low stoichiometry modifications. *N*-termini for the *S*-layer protein were especially varied, although all were consistent with signal peptide cleavage after residue 24. By abundance, the major *N*-terminal peptide observed was ADVIEIR, although peptides 14, 40, and 42 Da heavier were also found. The modified peptides followed ADVIEIR in elution by <1, 8, and 3 min, respectively.

*N*-terminal addition of 42 Da, localized by *y*_1_−*y*_6_ and *b*_2_ ions, (see Francoleon et al., 2009, **Figures 3C,D**) was initially attributed to α-amino acetylation. However, none of the tandem mass spectra acquired for this precursor yielded a *b*_1_-ion, generally considered diagnostic of *N*-terminal acetylation (Yalcin et al., 1995). Careful mass measurements on *b*_2_ product ions better matched modifications of composition C_2_H_2_O, rather than C_3_H_6_. The *M. acetivorans* ortholog, MA0829, also displayed evidence of signal peptide cleavage, yielding the *N*-terminal tryptic peptides VDVIEIR and a +42 Da variant, similarly lacking its *b*_1_ product ion by MS/MS (Francoleon et al., 2009 see Francoleon et al., 2009, **Figures 3A,B**). Further investigation is required to confidently ascribe *N*-terminal modifications for these low abundance variants.

MS/MS spectra suggest that the *M. mazei* +40 Da modification also localizes to the *N*-terminus, as *y*_1_−*y*_6_, *a*_2_, and *b*_2_−*b*_5_ ions were observed. Again, the *b*_1_-ion was not seen. The late elution of the +40 Da species relative to unmodified ADVIEIR, leads us to questions whether it might arise from in-source collision-induced dissociation (CID) of an exceptionally labile, hydrophobic group, leaving behind only a residual −N = CH-CH = O, −N = C(CH_3_)_2_, or −N = CH-CH_2_-CH_3_ *N*-terminus. Available mass accuracy narrowed consideration to the latter two possibilities. As an alternative to production by CID, the C_3_H_4_ increment could correspond to a Schiff base formed by addition of propionaldehdye, although the mechanism for such a modification is unclear. Further effort is required to characterize this C_3_H_4_ modification.

In some MM1976 ADVIEIR peptides, Glu_5_ was incremented by 14 Da, consistent with methyl esterification. Elsewhere, artifactual methyl adducts were attributed to incubation in acidic methanol during gel fixation or staining (Parker et al., 1998; Xing et al., 2008). Here the analyses displaying the modification were performed on proteins digested in solution; i.e., not subjected to staining. Their only exposure to methanol was shorter, at lower concentration and at lower temperature than conditions known to esterify. Thus, the 14 Da adducts are unlikely to be artifacts. Other instances of relevant methyl esters have been described previously (Hoelz et al., 2006).

### Other modified proteins

The predicted *N*-terminal peptide for MM1540, subunit *H* of tetrahydrosarcinopterin *S*-methyl transferase (MtrH) was confirmed to be MFKFDKKQE. Interestingly, peptide M. ASAWDWLR (residues 232–239) was also observed, potentially reflecting protein processing, anomalous cleavage, or alternate initiation, although we could find no rationalization for the latter possibility. That MM1540 is the catalytic subunit of the *S*-methyl transferase and binds methyl tetrahydrosarcinopterin (Hippler and Thauer, 1999) encourages speculation that an inadvertent methyl transfer to Met_231_ instead of the coenzyme M thiol might lead to a sulfonium-activated cyanogen bromide-like cleavage at the observed position.

**Figure 2:**
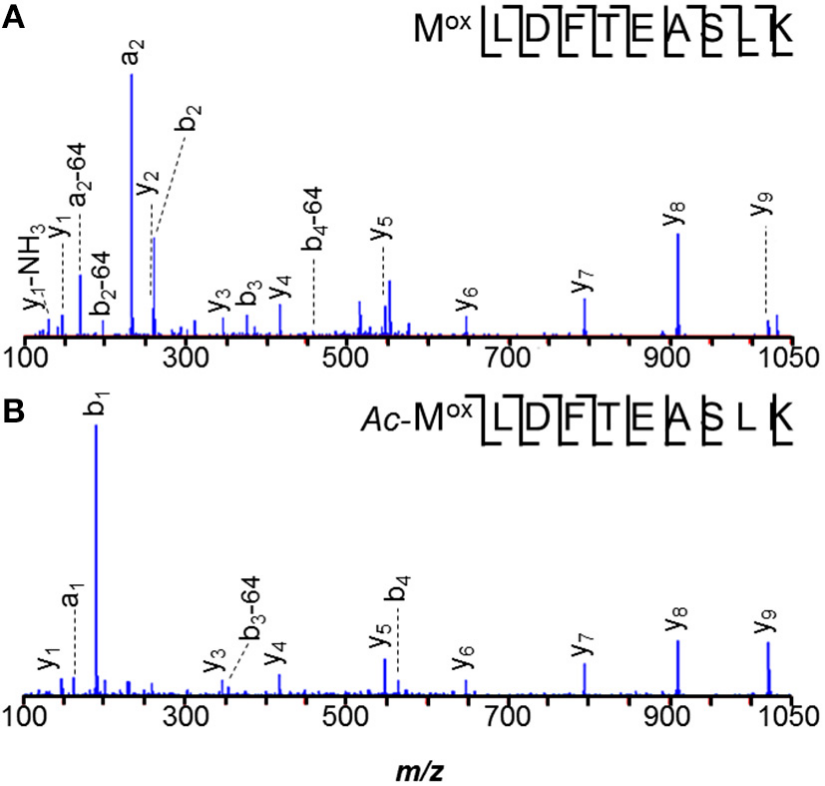
***N*-terminal peptides of *M. acetivorans* MA0456 (MtaC1). (A)** Unmodified MLDFTEASLK, **(B)** Low abundance variant Ac-M^ox^LDFTEASLK. Intense 190-Da ions correspond to the *b*_1_ product from the *N*-terminally acetylated peptide. Larger *b*-ions show 64-Da neutral loss products characteristic of methionine sulfoxide. [Based on its measured mass, the product ion observed at *m/z* 129.11 is attributed to *y*_1_–H_2_O (*m/z* 129.10), rather than unmodified *b*_1_ (*m/z* 129.01)].

The *N*-terminal peptide of *M. acetivorans* C2A MA0456 (MtaC1, methanol-5-hydroxybenzimidazolyl cobamide co-methyl transferase) was previously recovered as MLDFTEASLK and in its methionine sulfoxide form. Methionines are often oxidized under experimental conditions. A related peptide 58-Da heavier than the unmodified species was also observed (Figure 2). Tandem MS of the latter peptide localized the +58-Da to the first residue, revealing intense 190- and 126-Da product ions, consistent with the *b*_1_ ion from an *N*-terminally acetylated peptide and a corresponding 64-Da neutral loss product, unique to methionine sulfoxide. As discussed earlier, *b*_1_ observations strongly suggest *N*-terminal acetylation. Larger *b*-ions also showed 64-Da neutral loss products, establishing the variant *M. acetivorans* peptide as Ac-M^ox^LDFTEASLK. Peptide Ac-M^ox^LDFTEASLKK, was also observed. Previous mass analyses of intact *M. acetivorans* proteins reported *only* the free amino terminal form (Patrie et al., 2006). Interestingly, we observed only non-acetylated MLDFTEASLK from the *M. mazei* ortholog, MM1648, despite the larger protein quantity available for analysis. Note that *M. mazei* initiates translation 10 residues downstream of the originally annotated position) (Deppenmeier et al., 2002).

**Figure 3:**
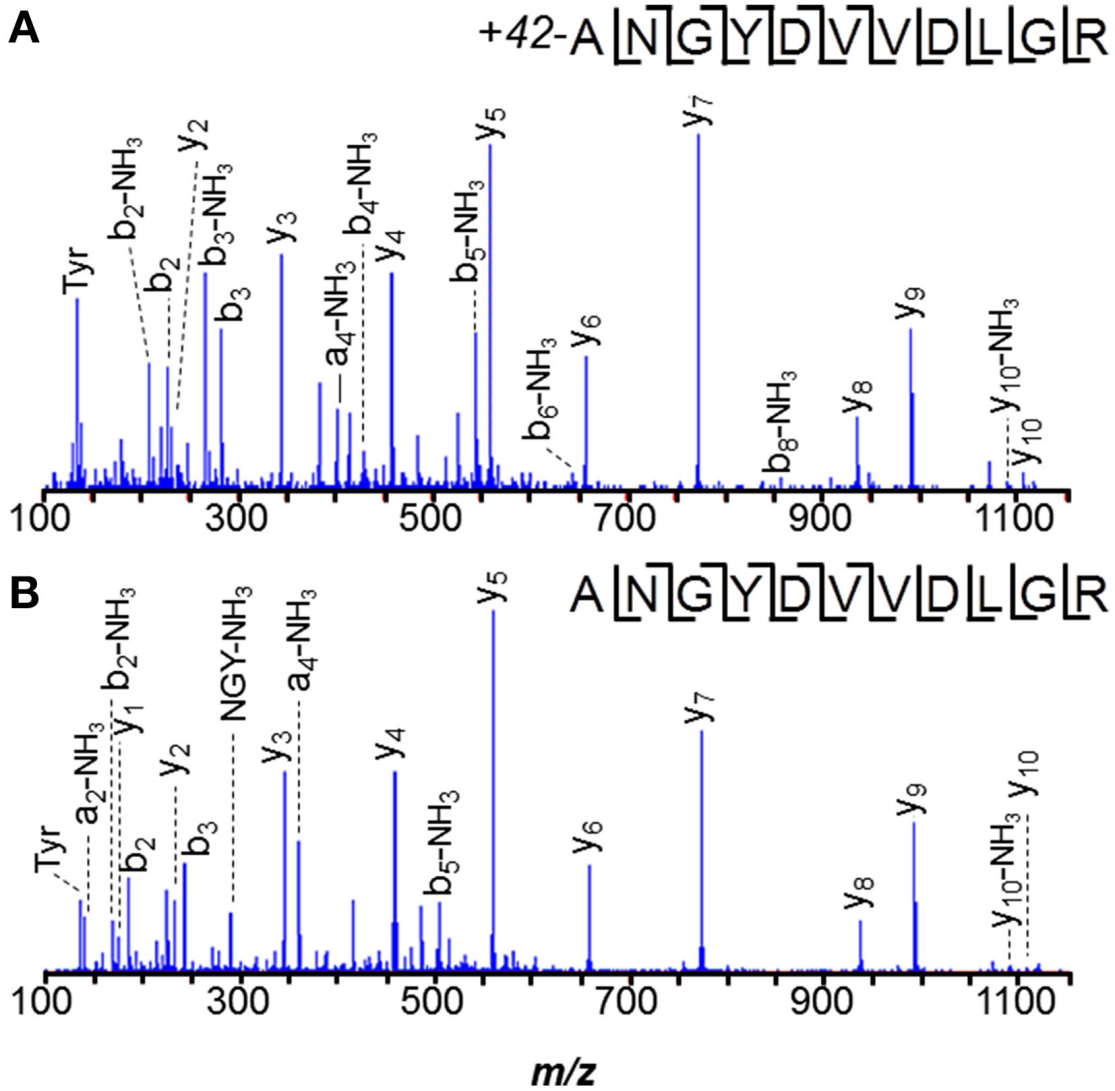
**Peptide ANGYDVVDLGR of *M. acetivorans* protein MA0456 (MtaC1) is present in two forms. (A)** Peptide with *N*-terminal +42-Da modification, consistent with trimethylation or a C_3_H_6_ composition. **(B)** Unmodified peptide.

Careful data mining revealed additional modifications to MA0456 (MtaC1). Three versions of peptide 149–159 were found: ANGYDVVDLGR, the Asn_2_ deamidated peptide, and a peptide 42-Da heavier than predicted, with its modification localized to the first residue by *y*_10_ and *b*_2_ ions (Figure 3). The absence of a *b*_1_-ion and accurate mass measurement ruled out *N*^α^-acetylation for the heavier peptide, but could support an *A*→*I/L* substitution or *N*^α^-trimethylation. However, amino acid substitution is hard to rationalize from the nucleotides coding Ala (GCA) vs. those coding Leu/Ile (CTX, TTA, TTG/ATT, ATC, ATA), as is sub-stoichiometric substitution. *N*^α^-trimethylation would require prior cleavage of the protein to expose *N*-terminal ANGYDVVDLGR for modification. Interestingly, the peptide lies near His_136_, an axial ligand to the Co^2+^ of the MtaC1 corrinoid co-factor that accepts CH_3_ from methanol and subsequently transfers it to coenzyme M (Sauer et al., 1997; Randaccio et al., 2007). Neutral losses of 59-Da, sometimes observed from trimethylated residues, were not apparent in these spectra. Additional experiments are required to verify the source of this substitution; e.g., genetic drift (DNA), sloppy transcription (RNA), miscoding (translation), or post-translational modification, but at present trimethylation is favored.

**Figure 4:**
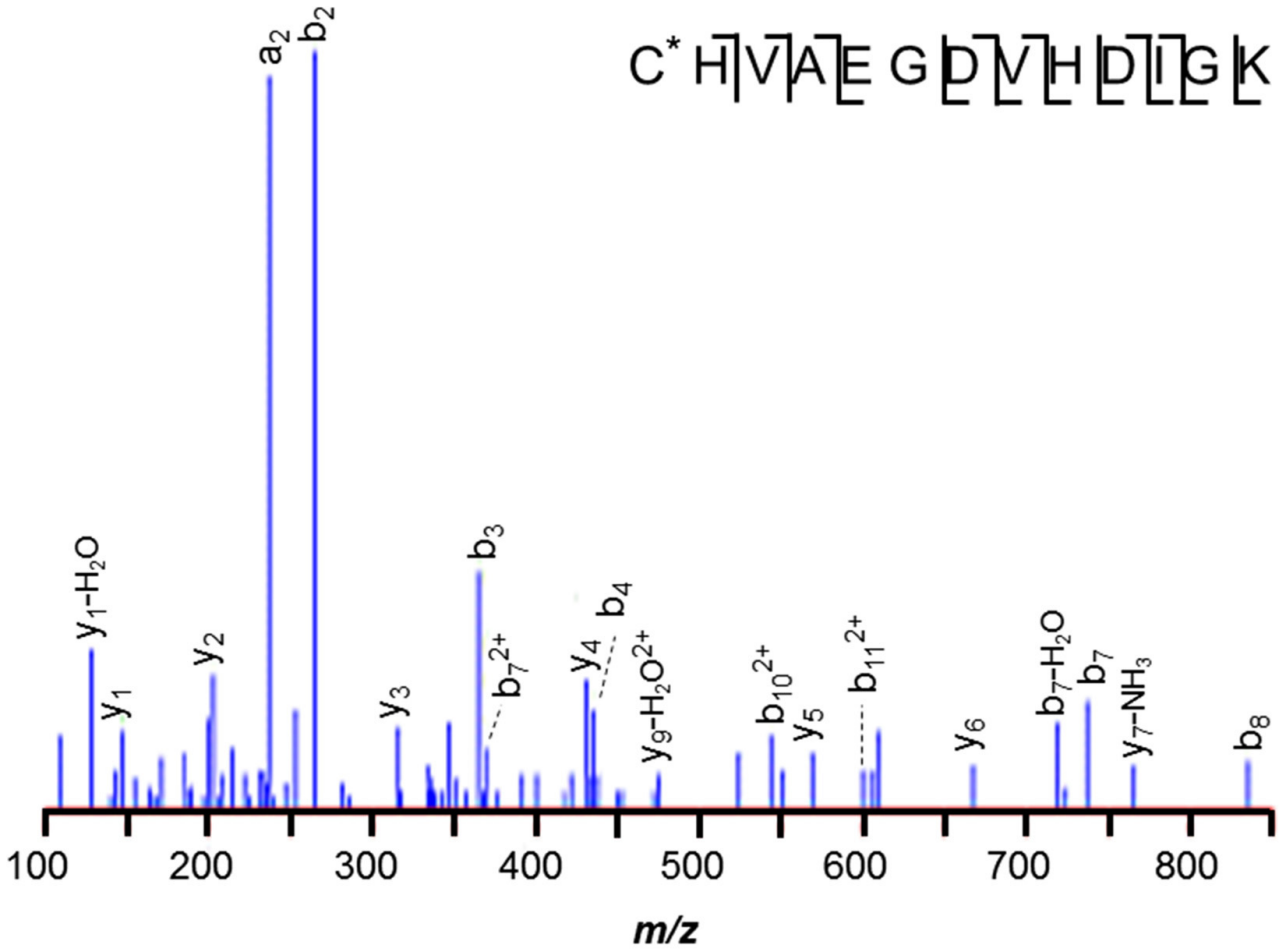
***M. mazei* semi-tryptic peptide from MM1073 (MtaC2)**. Peptide 138–150, C^*^HVAEGDVHDIGK is incremented by 25-Da at its *N*-terminus, consistent with cyanylation. The sequence is common to segment 138–150 of methanol corrinoid proteins MM1648 (MtaC1) and MM1073 (MtaC2).

Despite the larger protein amounts available for the two *M. mazei* orthologs, MM1648 (MtaC1) and MM1073 (MtaC2), 42-Da incremented peptides ANGYNVVDLGR and ANGYDVVDLGR, respectively, were not observed. Only unmodified and -17 Da variants were observed, with the latter reflecting succinimides, unremarkable for Asn-Gly bonds. Instead, a semi-tryptic peptide was observed in *M. mazei* MM1073 (MtaC2). Peptide 138–150, C^*^HVAEGDVHDIGK was incremented by 25-Da at its *N*-terminus, consistent with cyanylation see the MS/MS spectrum displayed in Figure 4). Such modification seems remarkable, but may reflect a radical-induced side reaction given that this region binds the corrinoid cofactor. A classic chemical cleavage scheme relies on *S*-cyanocysteine's base-catalyzed ability to cleave the *N*-terminal peptide bond to yield iminothiazolidinyl peptides (Jacobson et al., 1973; Degani and Patchornik, 1974; Nefsky and Bretscher, 1989; Wu and Watson, 1997). Uncleaved *S*-cyanocysteine 134–150 (GTVVC^*^HVAEGDVHDIGK) was not observed by LC-MS. At present, we cannot differentiate *in vivo* modification from modification induced by exposure to air during sample handling.

Limited experiments performed on *M. acetivorans* MA0456 (MtaC1), revealed only unmodified tryptic peptide GTVVCHVAEGDVHDIGK (124–140), but those studies cannot be considered conclusive, because smaller quantities of protein and a narrower range of sample handling conditions were pursued. In particular, the *M. acetivorans* samples were reduced with dithiothreitol (DTT), whereas cyanylated *M. mazei* protein was observed from untreated samples. Excess DTT removes the cyanide group from internal cysteines, thereby stopping the cleavage reaction (Degani and Patchornik, 1974; Nefsky and Bretscher, 1989).

### Protein complexes for energy transfer-acquisition

Numerous protein complexes were recovered by Con A fractionation, including, tetrahydromethanopterin *S*-methyl transferase (*mtr*), methylcobalamin:CoM methyltransferase (*mta*), methyl CoM reductase (*mcr*), F_420_H_2_ dehydrogenase (*fpo*), heterodisulfide reductase (*hdr*), and A_1_A_0_ H^+^ ATPase (*aha*). Conceivably, Con A fractionation could show utility for enriching select *M. mazei* protein complexes in support of other studies or for additional characterization. Hence, we considered which subunits were identified in the eluate. A second reason for interest in the proteins and complexes detected is that mass spectrometrists often discount the presence of certain proteins in mixtures as reflective of contamination. These assumptions are not always justified. Here we sought evidence that proteins not annotated as surface- or membrane-localized, or not known to be glycosylated might rationally be carried along in the Con A fractionation by interactions with other proteins. Should most of the detected proteins be rationalized, there would be additional impetus to explore the cellular localization of any remaining proteins.

All eight tetramethyl methanopterin *S*-methyltransferase subunits (MtrABCDEFGH) were detected, including integral membrane proteins MtrC, MtrD, and MtrE (Fischer et al., 1992; Lienard et al., 1996; Lienard and Gottschalk, 1998; Thauer, 1998; Kahnt et al., 2007). The abundance of this complex enabled *N*- and *C*-termini to be defined for many of the subunits, as well as providing some unexpected observations. Tryptic peptides IVTDEDKGIFDR (40–51) and IVTD**X**DK (40–46), with *X* corresponding to Glu-28 Da, were observed from catalytic subunit MtrH (MM1540). The latter peptide could reflect decarboxylation of Glu_44_, perhaps from exposure to reactive species.

Strikingly, Con A binding recovered representatives from all types of the organism's membrane-bound hydrogenases involved in electron transfer: the F_420_ non-reducing hydrogenase (Vht), Ech hydrogenase, and the F_420_H_2_ dehydrogenase (Fpo) (Künkel et al., 1997, 1998). These included seven proteins encoded in the F_420_H_2_ dehydrogenase gene cluster MM2479-2491, the hydrogenase components EchA and EchB (MM2320, MM2321), and F_420_–non-reducing hydrogenase MM2171 (VhtC). Previously, we recovered a large subunit of the F_420_H_2_ dehydrogenase [MM2170 (VhtA) or MM2313] by biotin-tagging (Francoleon et al., 2009). Membrane-bound heterodisulfide reductase proteins HdrE and HdrD (MM1843 and MM1844) were also recovered, as was MM0628, provider of reduced F_420_ (Bäumer et al., 2000; Thauer et al., 2008).

**Figure 5:**
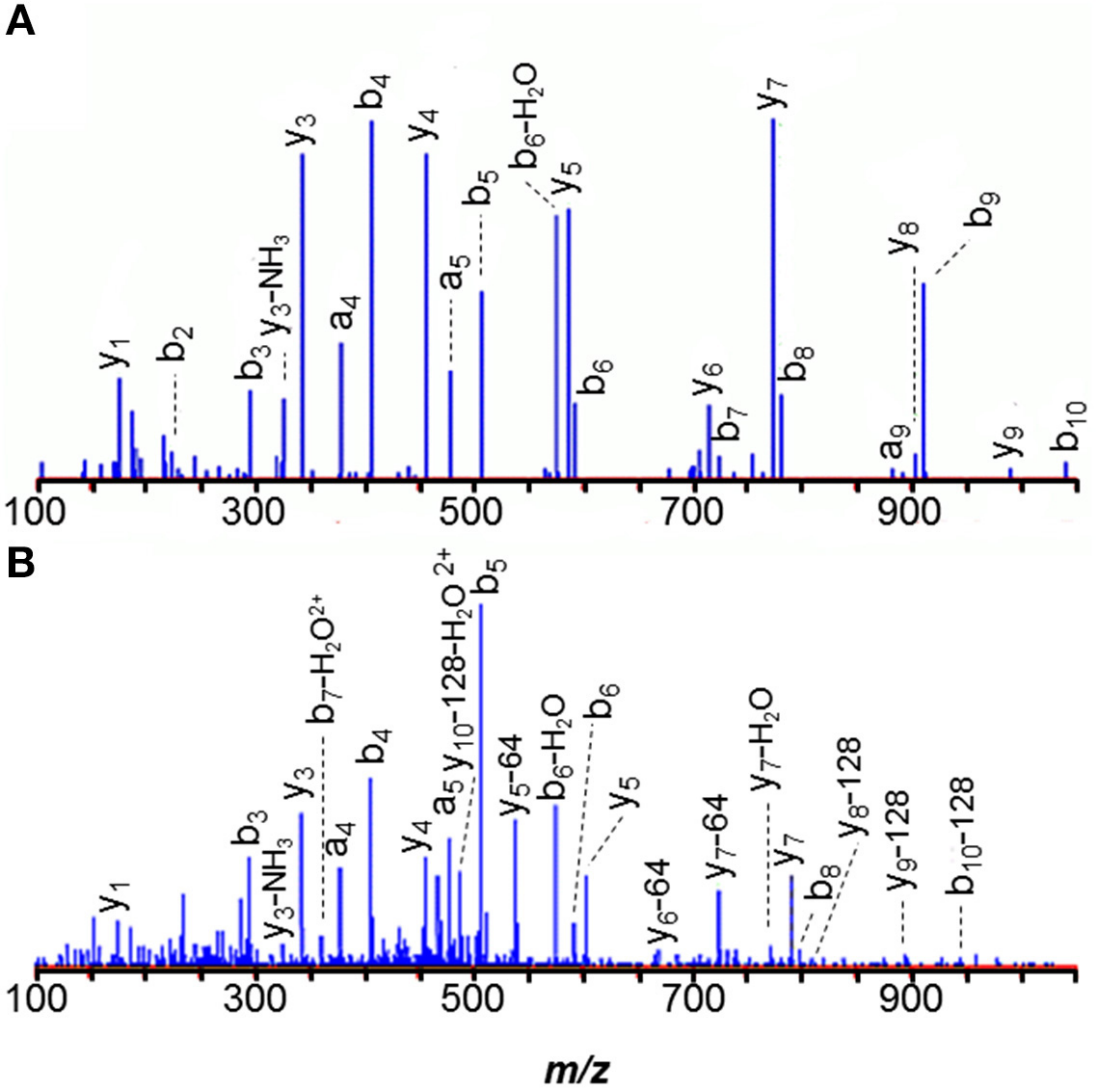
**MS/MS spectra of the 271–284 tryptic peptides from the alpha subunit of membrane-associated methyl coenzyme M reductase (McrA) show that the 1-*N*-methyl histidine residue signals its presence with strongly enhanced *b*-ion intensities. (A)** *M. mazei* MM1240 H^*^AALVSMGEMLPAR, and **(B)** *M. acetivorans* MA4546 H^*^AALVSM^ox^GEM^ox^LPAR.

The methyl coenzyme M product (CH_3_-S-CoM) is reductively demethylated by coenzyme B (CoB-SH) in a process catalyzed by the membrane-associated methyl coenzyme M reductase complex, Mcr (Hoppert and Mayer, 1990). All three α, β, and γ subunits (MM1240, McrA; MM1244, McrB; MM1241, McrC) were eluted from concanavalin A. From α-subunit McrA, 1-*N*-methyl-histidine was observed in peptide HAALVSMGEMLPAR (271–284), consistent with previous observations in *M. barkeri* (Grabarse et al., 2000). Because peptides spanning residues 285, 465, and 472 were not recovered, we could not determine if the unusual amino acids *5*-methylarginine, *S*-methylcysteine, and thioglycine were present, as in other methanogens (Grabarse et al., 2000; Kahnt et al., 2007). However, the *N*-terminus was found to be acetylated. The peptide 271–284 MS/MS spectrum, illustrated in Figure 5A, demonstrates that the *1*-*N*-methyl histidine residue enhances *b*-ion intensities from tryptic peptides. Peptides 271–284 and 1–7 were only observed in modified form. Similarly, *1-N*-methyl histidine was found in the active site region of the *M. acetivorans* ortholog, MA4546. (See Figure 5B).

That protein products from 8 of 10 A_1_A_0_ ATPase-related genes were recovered *via* Con A elution, (*ahaABCDE, ahaHIK*) suggests that this capture method may be useful in isolating these unstable complexes (Müller et al., 1999). Previous experiments (Lemker et al., 2001, 2003) purifying ATPase subcomplexes from F_1_F_0_ ATPase-negative *E. coli* cells over-expressing the *M. mazei ahaA-ahaG* operon recovered subunits A, B, C, D, and F. Questions persist regarding the participation in ATPase of *M. maze*i AhaG, considered authentic based on observation of an appropriately migrating SDS-PAGE band following heterologous expression in *Escherichia coli* (Lemker et al., 2001), but homologs of which are absent in several archaea. In our studies, recovery of AhaG may have been reduced, because the small (6.3-kDa) protein is expected to yield only two tryptic peptides larger than 900 Da, one of which is very hydrophobic. Although the mass spectrometer is capable of detecting tryptic peptides below 900 Da in mass, the peptides are often lost upstream in reversed phase HPLC, because their hydrophilicity causes them to elute with salt. Large hydrophobic peptides are also problematic for the chromatography because they fail to elute during the analysis. Thus, we cannot rule out AhaG as a component of the A_1_A_0_ ATPase.

### Protein complexes for methanol metabolism

Observed in these studies were products from two of the three operons coding methanol-specific methyl cobalamin:CoM methyltransferases: (1) MtaA1 (MM1070) with heterodimeric MtaB2/MtaC2 (MM1074/MM1073), and (2) MtaB1/MtaC1 (MM1647/MM1648). The MtaB/MtaC complexes transfer a methyl group from methanol to the corrinoid cofactor of MtaC. Subsequently, that methyl is transferred to coenzyme M (HS-CoM), catalyzed by MtaA1. Alternative enzymes catalyzing transfer to HS-CoM (MtaA2 and MtbA), were not observed. That MtbA was absent is unsurprising, as it is specific for growth on H_2_/CO_2_ or trimethylamine (Harms and Thauer, 1996; Hovey et al., 2005), and Ding et al. (2002) did not identify ortholog MtaA2 from methanol-cultivated *M. thermophila*. However, methanol-induced expression of MtaB3 and MtaC3 was established in *M. thermophila* (Ding et al., 2002), leading us to address their absence in our data. First, we would not expect these methyl transferases to bind concanavalin A or associate with Con A binders, because MtaB3 and MtaC3 are generally considered *soluble*. Also, several MtaB3 (MM0175) peptides are non-unique, (shared with other isozymes) complicating its identification from complex mixtures. DNA microarray analyses indicated that *mtaB1/mtaC1* were induced 10–33X in methanol, while *mtaB2/mtaC2* were induced only in acetate (Hovey et al., 2005). Quantifying roughly, by comparing numbers of peptides recovered, we see that the trend in protein abundances follows the same direction as the transcripts: 14 peptides vs. 9 for MtaB1 vs. MtaB2 and 10 peptides vs. 6 for MtaC1 vs. MtaC2.

### Additional proteins recovered by Con A

MM0633, a hypothetical protein containing a multi-heme cytochrome *c* domain suggested as part of a membrane-bound complex, belongs to a gene cluster showing elevated expression under aceticlastic growth (Hovey et al., 2005). In these methylotrophic studies, however, MM0633 was the only cluster member observed. As it lacks transmembrane regions, we may wonder if its presence reflects interaction with some other membrane protein or glycosylation.

The oligosaccharyl transferase (MM0647) detected in the ConA pull down experiment (Table 1) is a product of one of three *aglB* homologs encoded in the *M. mazei* genome (MM646, MM0647, MM2210) (Magidovich and Eichler, 2009). Its detection makes MM0647 a logical candidate for the AglB oligosaccharyl transferase that links glycans to asparagines on surface layer protein MM1976 and on other *N*-linked glycoproteins. Two minor *S*-layer proteins similar to MM1976, MM0467, and MM1364, were identified where the latter was also shown to be glycosylated (described below). Ongoing analyses of *M. mazei N*-linked glycans will reveal more about the oligosaccharides transferred.

Proteins with roles in cobalt and iron uptake were also observed: MM2069, an iron ABC transporter, MM1999 and MM2000 involved in cobalt uptake, MM0893 (CbiM), a cobalt ATP-dependent transporter, and MM0994 (CbiC). Identifications for CbiC and CbiM were based on a single recovered peptide for each, a lower standard of confidence. Numerous other transporters were also observed.

Previously detected Hsp70 analog MM2505 and the membrane-bound ATP-dependent protease LonB were also found in Con A eluate, along with two of three subunits comprising the *M. mazei* thermosome (Bateman et al., 2004), a eukaryotic-type chaperonin complex. Previously, surface-biotinylation with streptavidin affinity chromatography retrieved all 3 *M. mazei* subunits [MM1379 (α), MM0072 (β), and MM1096 (γ)], confirming the proposal (Trent et al., 2003) that a fraction of thermosome (or rosettasome) complexes are membrane-localized. In the present *M. mazei* lectin capture, as well as for our previous *M. acetivorans* surface-tagging and capture efforts (Francoleon et al., 2009), the thermosome γ-subunit was not recovered. Archaeal thermosomes vary in whether their double ring structures are composed of identical subunits, or of two or three different sequences; e.g., the *Methanopyrus kandleri* complex is homomeric (Andrä et al., 1998). Indeed, *M. mazei* proteins most closely related to the *M. kandleri* thermosome, MK1006, are MM1379, MM1096, and MM0072, respectively.

### Protein export and processing

Machinery to transport proteins across the membrane is essential to protein secretion. Recent cryo-electron microscopy studies revealed important components of this machinery in yeast, where protein transport across the endoplasmic reticulum begins with the signal peptide of the nascent chain engaging the signal recognition particle (SRP) in the cytoplasm. Co-translational translocation is initiated when the signal peptide is transferred to the protein conducting channel, overlapping 4 binding sites on the large ribosomal subunit. The archaeal analog to translocation across the ER is transport across the cell membrane. The hetero-trimeric protein-conducting channel (akin to yeast Sec61α, β, and γ) consists of integral membrane proteins: MM2147, MM1372, and MM1009, respectively, all of which we observed (Becker et al., 2009; Kampmann and Blobel, 2009), along with accessory factors MM1424 (SecF) and MM1425 (SecD). As in Eukarya and Bacteria, ribosomes contact membranes *via* Sec-based sites (Ring and Eichler, 2004), consistent with our observations of associated ribosomal proteins. The SecP algorithm predicts secretion of ribosomal proteins MM1760, MM2124, MM2135, and MM2157 (Bendtsen et al., 2004a, 2005), although the relationship between non-classical secretion predictions, disordered regions, and protein-protein or protein-nucleotide interactions is yet unclear. The mass spectrometry/proteomics community often cites the presence of ribosomal proteins in cell membrane preparations as evidence of poor quality, but it is important to consider that some presence in preparations enriching membrane-*associated* complexes is legitimate.

Of the total number of *M. mazei* open reading frames detected by our study, genome sequence analysis (Deppenmeier et al., 2002) annotated about 20% as hypothetical, thus highlighting the efficacy of the Con A pull-down approach for discovery. Of the 28 hypothetical proteins, 24 were predicted to be secreted by Exprot, SignalP, SecP, and/or LipoP (Juncker et al., 2003; Bendtsen et al., 2004a,b, 2005; Saleh et al., 2010). In addition, we find that hypothetical proteins MM0716 and MM1364 are glycosylated. Glycosylation of MM1364 correlates with homology to known *Methanosarcinae S*-layer proteins (Francoleon et al., 2009), that also bear glycans.

## Conclusions

LC-MS/MS analyses of proteolytically-digested concanavalin A eluate from *M. mazei* Gö1 cell lysates led to the identification of 154 proteins. Among these, constituents of membrane-bound or membrane-associated complexes known from the literature were well-represented, including all 8 subunits of tetrahydromethanopterin S-methyl transferase (Mtr), seven proteins encoded by the F_420_H_2_ dehydrogenase *fpo* operon, the 3 subunits of methyl coenzyme M reductase (Mcr), the protein products from 8 of 10 A_1_A_0_ ATPase-related genes (*ahaABCDEHIK*), and components of the machinery translocating proteins across the cell membrane [protein channel constituents MM2147, MM1372, and MM1009 and accessory factors MM1424 (SecF) and MM1425 (SecD)]. All of these proteins do not bear Con A-interacting saccharides, because lectin binding is performed under non-denaturing conditions. However, the results can be useful in considering strategies to enrich or isolate select membrane complexes from *M. mazei*, and perhaps other *Methanosarcinae*, in order to monitor dynamic changes in protein modifications and/or retrieve complexes from strains not engineered to synthesize tagged proteins for easy retrieval.

Tandem mass spectrometry data associated with protein identifications can be mined to recover novel information that is not automatically provided by the high-throughput analyses. Here it was found that *S*-layer protein MM1976 was present in multiple forms, including four variants of its *N*-terminal peptide ADVIEIR. Instances of protein formylation, methyl esterification, methylation, and cyanylation were also found. Knowledge of unanticipated modifications, even if not providing immediate insight, does suggest features to monitor for evidence of dynamic changes. Knowledge gained by data mining can also complement what is obtained from experiments specifically targeting that modification, because the experimental conditions (e.g., chromatography resin and elution conditions) are often different. A high throughput LC-MS/MS run injects only a few hundred nanograms of a peptide mixture. Further effort is underway to characterize unknown glycans, the sites they modify, and other post-translational modifications, particularly in extensively modified *S*-layer protein MM1976.

*N*-termini recovered from a subset of proteins secreted to the membrane or cell surface provide a dataset for comparison to signal peptide algorithms. Disagreement between the number of proteins predicted vs. detected with signal peptide-excised *N*-termini suggests that leaderless secretion is of greater importance than present models imply.

### Conflict of interest statement

The authors declare that the research was conducted in the absence of any commercial or financial relationships that could be construed as a potential conflict of interest.
