# Supplementary material for: Mining proteomic data to expose protein modifications in Methanosarcina mazei strain Gö1
Source: Front Microbiol. 2015 Mar 5;6:149. doi: 10.3389/fmicb.2015.00149 (PMC4350412; doi:10.3389/fmicb.2015.00149)
Supplement: Supplementary file 1 [file Table1.DOCX]

**Table S-1** Proteins Detected by a Single Peptide in Concanavalin A Eluate

| ***Uniprot Accession*** | ***MM#*** | ***Name*** | ***Mascot Protein Score*** | ***Exprot^a^*** | ***SignalP^b^*** | ***SecP^c^*** | ***LipoP^d^*** |
| --- | --- | --- | --- | --- | --- | --- | --- |
| Q8Q0X6 | MM0004 | DppB | 46 |  | Y | Y |  |
| Q8Q0S3 | MM0063 | SLB, surface layer protein B | 31 | 1 |  | Y |  |
| Q8PZY5 | MM0353 | Hypothetical protein | 26 | 1 |  | Y |  |
| Q8PZW4 | MM0375 | Hypothetical protein | 32 |  | Y* | Y |  |
| Q8PZV8 | MM0381 | Peptidyl-prolyl cis-trans isomerase | 60 |  |  | Y |  |
| Q8PZH9 | MM0514 | NifH2 | 47 | 2 |  |  |  |
| Q8PZ19 | MM0676 | F390 synthetase | 40 |  |  |  |  |
| Q8PYS8 | MM0774 | Hypothetical protein | 59 |  |  |  |  |
| Q60188 | MM0778 | AtpD | 52 |  |  |  |  |
| Q8PYG9 | MM0893 | CbiM | 38 |  |  | Y |  |
| Q8PYF4 | MM0908 | ABC Transporter, hypothetical | 76 |  | Y* | Y |  |
| Q8PYE3 | MM0919 | Peptidase, hypothetical | 38 |  | Y* | Y |  |
| Q8PY77 | MM0987 | Conserved protein | 35 |  |  |  |  |
| Q8PY70 | MM0994 | Precorrin-8X methyl mutase, CbiC superfamily | 39 |  |  |  |  |
| Q8PY61 | MM1003 | Isocitrate dehydrogenase | 60 |  |  |  |  |
| Q8PY56 | MM1008 | FtsZ | 115 |  |  |  |  |
| Q8PY55 | MM1009 | SecE | 59 |  |  | Y |  |
| Q8PY50 | MM1014 | L12AE LSU ribosomal protein | 122 |  |  |  |  |
| Q8PXV2 | MM1114 | Hypothetical protein | 29 |  |  |  |  |
| Q8PXQ8 | MM1158 | Hypothetical protein | 22 |  |  | Y |  |
| Q8PXJ7 | MM1221 | Mc1 chromosomal protein | 56 | 1 |  | Y |  |
| Q8PXJ6 | MM1222 | MptA, GTP cyclohydrolase | 54 |  |  |  |  |
| Q8PXE8 | MM1272 | DhqS, 3-dehydroquinate synthase | 85 |  |  |  |  |
| Q8PXB7 | MM1305 | Hypothetical protein | 33 |  |  |  |  |
| Q8PX93 | MM1329 | Methyl-accepting chemotaxis protein | 79 |  | Y |  |  |
| Q8PX69 | MM1353 | 5-Methyltetrahydropteroyl-triglutamate-homocysteine methyltransferase | 42 |  |  |  |  |
| Q8PX50 | MM1372 | SecG | 78 |  |  | Y |  |
| Q8PX34 | MM1388 | Glycyl-tRNA synthetase | 37 |  | Y* |  |  |
| Q8PWW2 | MM1464 | Hypothetical protein | 48 |  | Y* | Y |  |
| Q8PWU7 | MM1479 | Helicase | 36 |  |  |  |  |
| Q8PWJ5 | MM1590 | Hypothetical protein | 54 |  |  |  |  |
| Q8PWG0 | MM1629 | Transporter, hypothetical | 33 | 1 |  | Y |  |
| Q8PWC0 | MM1669 | HsdM, type I restriction-modification system methyltransferase subunit | 29 |  |  |  |  |
| Q8PW51 | MM1750 | Hypothetical protein | 40 | 1 | Y | Y |  |
| Q8PW45 | MM1756 | Rpl13p | 28 |  |  |  |  |
| Q8PW34 | MM1767 | Dimethylallyltransferase | 62 |  |  |  |  |
| Q8PVW3 | MM1844 | HdrD | 48 |  |  |  |  |
| Q8PV50 | MM2124 | Rpl3p | 67 |  | Y | Y |  |
| Q8PV44 | MM2130 | Rps3p, 30S S3p, | 51 |  |  |  |  |
| Q8PV41 | MM2133 | Rps17p, 30S S17 | 30 |  |  |  |  |
| Q8PV39 | MM2135 | RL24 | 61 |  |  | Y |  |
| Q8PV38 | MM2136 | RS4e | 22 |  |  |  |  |
| Q8PUL3 | MM2321 | EchB, EcH hydrogenase | 33 |  | Y | Y | Y |
| Q8PU77 | MM2464 | NDK | 78 |  |  |  |  |
| Q8PU74 | MM2467 | Rpl7ae | 66 |  |  |  |  |
| Q9P9G0 | MM2488 | FpoD | 64 |  |  |  |  |
| Q8PU58 | MM2491 | FpoA | 84 |  |  | Y |  |
| Q8PU43 | MM2513 | Hypothetical protein | 51 | 1 |  | Y |  |
| Q8PTU1 | MM2620 | PsmA, proteasome, alpha subunit | 40 |  |  |  |  |
| Q8PTR0 | MM2653 | Mch | 42 |  |  |  |  |
| Q8PTK5 | MM2710 | Hypothetical protein | 21 | 1 | Y | Y |  |
| Q8PT29 | MM2889 | Hypothetical protein | 25 |  | Y* | Y |  |
| Q8PSV0 | MM2976 | Type 1 restriction system | 31 |  |  |  |  |
| Q8PSM3 | MM3056 | ComA, competence-like protein | 25 |  |  | Y |  |

a) Exprot (Saleh et al., 2010): 1=Predicted type I signal peptidase substrate; 2=Predicted type II signal peptidase substrate

b) SignalP (Bendtsen et al., 2004b): Y=Predicted signal peptide, SignalP 3.0

c) SecP (Bendtsen et al., 2004a;Bendtsen et al., 2005): Y=Predicted substrate for leaderless secretion, SecP 2.0

d) LipoP (Juncker et al., 2003): Y=Predicted lipoprotein

* Predicted as secreted only by the SignalP eukaryotic predictor
