# Supplementary material for: Mining proteomic data to expose protein modifications in Methanosarcina mazei strain Gö1
Source: Front Microbiol. 2015 Mar 5;6:149. doi: 10.3389/fmicb.2015.00149 (PMC4350412; doi:10.3389/fmicb.2015.00149)
Supplement: Supplementary file 2 [file Table2.DOCX]

**Table S-2** Predicting Signal Peptides, Transmembrane Helices, Lipid-Addition, and Non-Classical Secretion for Proteins Having Detected *N-*termini

| ***Accession*** | ***MM#*** | ***SignalP*** | ***TMH*** | ***LipoP*** | ***Sec P*** | ***Exprot*** |
| --- | --- | --- | --- | --- | --- | --- |
| F1SVJ4 | MM0784 | Y* | 2 |  | Y | 1 |
| Q8PYJ5 | MM0866 |  | 1 |  | Y | 1 |
| Q8PY55 | MM1009 |  | 1 |  | Y |  |
| Q8PXZ3 | MM1073 |  | 0 |  |  |  |
| Q8PXZ2 | MM1074 |  | 0 |  |  |  |
| Q8PXZ1 | MM1075 |  | 6 |  | Y | 1 |
| Q8PXX0 | MM1096 |  | 0 |  |  |  |
| Q8PXV8 | MM1108 |  | 0 |  |  |  |
| Q8PXJ7 | MM1221 |  | 0 |  | Y | 1 |
| Q8PXH8 | MM1240 |  | 0 |  |  |  |
| Q8PXH4 | MM1244 |  | 0 |  |  |  |
| Q8PXG7 | MM1251 | Y* | 10 |  | Y |  |
| Q8PX60 | MM1362 | Y | 0 |  | Y | 2 |
| Q8PX43 | MM1379 |  | 0 |  |  |  |
| Q8PWZ8 | MM1424 | Y | 6 |  | Y | 1 |
| P80650 | MM1540 |  | 0 |  |  |  |
| P80654 | MM1542 |  | 1 |  | Y |  |
| O59640 | MM1543 |  | 1 |  |  |  |
| P80655 | MM1544 |  | 1 |  | Y |  |
| P80651 | MM1547 | Y | 6 |  | Y |  |
| Q8PWE2 | MM1647 |  | 0 |  |  |  |
| Q8PWE1 | MM1648 |  | 0 |  |  |  |
| Q8PVI7 | MM1976 | Y | 2 |  | Y | 1 |
| Q8PUR8 | MM2264 |  | 0 |  |  |  |
| Q8PU74 | MM2467 |  | 0 |  |  |  |
| F1SVH9 | MM2481 | Y | 14 |  | Y |  |
| F1SVE0 | MM2483 | Y | 3 |  | Y |  |

SignalP 3.0 (Bendtsen et al., 2004b), LipoP (Juncker et al., 2003), and SecretomeP 2.0 (SecP) (Bendtsen et al., 2004a;Bendtsen et al., 2005) algorithms were employed to predict secreted proteins, while Exprot predictions for *M. mazei* were obtained from supplemental information in Saleh, *et al.* (Saleh et al., 2010). TMH refers to the predicted number of transmembrane helices.

* Predicted as secreted only by the SignalP eukaryotic predictor

Red entries correspond to identifications based on a single peptide.
