# Supplementary material for: Mining proteomic data to expose protein modifications in Methanosarcina mazei strain Gö1
Source: Front Microbiol. 2015 Mar 5;6:149. doi: 10.3389/fmicb.2015.00149 (PMC4350412; doi:10.3389/fmicb.2015.00149)
Supplement: Supplementary file 3 [file Table3.DOCX]

**Table S-3** Observed Proteins Predicted to be Secreted by SignalP 3.0 and their *N*-Glycosylation Sites Predicted by NetNGlyc 1.0 Server

| ***Uniprot Access.*** | ***MM#*** | ***Name*** | ***Prokaryotic Gram -*** | ***Prokaryotic Gram +*** | ***Eukaryotic*** | ***Glyco Sites*** |
| --- | --- | --- | --- | --- | --- | --- |
| Q8Q0X6 | MM0004 | DppB |  | 🗸 |  | 1 |
| Q8Q0S0 | MM0066 | Hypothetical protein | 🗸 | 🗸 | 🗸 | 23 |
| Q8PZW4 | MM0375 | Hypothetical protein |  |  | 🗸 | 9 |
| Q8PZM5 | MM0467 | *S*-layer paralog | 🗸 | 🗸 | 🗸 | 8 |
| Q8PYZ8 | MM0700 | HppA |  |  | 🗸 | 1 |
| Q8PYY3 | MM0716 | Hypothetical protein | 🗸 | 🗸 |  | 7 |
| F1SVJ4 | MM0784 | AtpK |  |  | 🗸 | 0 |
| Q8PYF4 | MM0908 | ABC transporter, hypothetical |  |  | 🗸 | 0 |
| Q8PYE3 | MM0919 | Peptidase, hypothetical |  |  | 🗸 | 0 |
| Q8PY73 | MM0991 | Thioredoxin | 🗸 | 🗸 | 🗸 | 5 |
| Q8PXI2 | MM1236 | HtpX protease | 🗸 | 🗸 | 🗸 | 0 |
| Q8PXG8 | MM1250 | Cation transporter |  |  | 🗸 | 2 |
| Q8PXG7 | MM1251 | Cation transporter |  |  | 🗸 | 2 |
| Q8PX93 | MM1329 | Methyl-accepting chemotaxis | 🗸 |  | 🗸 | 6 |
| Q8PX89 | MM1333 | Zinc ABC transporter | 🗸 | 🗸 | 🗸 | 2 |
| Q8PX60 | MM1362 | Putative aliphat. Sulfonate bind. | 🗸 | 🗸 | 🗸 | 4 |
| Q8PX58 | MM1364 | *S*-layer paralog | 🗸 | 🗸 | 🗸 | 13 |
| Q8PX34 | MM1388 | Glycyl-tRNA synthetase |  |  | 🗸 | 0 |
| Q8PWZ8 | MM1424 | SecF | 🗸 | 🗸 |  | 0 |
| Q8PWZ7 | MM1425 | SedD |  | 🗸 | 🗸 | 3 |
| Q8PWW2 | MM1464 | Hypothetical protein |  |  | 🗸 | 0 |
| P80653 | MM1546 | MtrD | 🗸 | 🗸 | 🗸 | 1 |
| P80561 | MM1547 | MtrE | 🗸 | 🗸 | 🗸 | 0 |
| Q8PWN5 | MM1549 | Na/proline symporter |  |  | 🗸 | 0 |
| Q8PWN3 | MM1551 | Hypothetical protein | 🗸 | 🗸 | 🗸 | 3 |
| Q8PWM3 | MM1561 | ABC transporter, W-binding |  |  | 🗸 | 4 |
| Q8PWJ6 | MM1589 | Surface layer protein B | 🗸 | 🗸 | 🗸 | 14 |
| Q8PWA1 | MM1695 | Hypothetical protein | 🗸 | 🗸 | 🗸 | 4 |
| Q8PW51 | MM1750 | Hypothetical protein | 🗸 | 🗸 | 🗸 | 2 |
| Q8PVY9 | MM1816 | Conserved Protein | 🗸 | 🗸 | 🗸 | 5 |
| Q8PVW4 | MM1843 | HdrE, heterodisulfide reductase | 🗸 |  | 🗸 | 0 |
| Q8PVV1 | MM1859 | DdpA, ABC transporter | 🗸 | 🗸 | 🗸 | 3 |
| Q8PVM4 | MM1939 | Glutamine binding Protein | 🗸 | 🗸 | 🗸 | 1 |
| Q8PVI7 | MM1976 | *S*-layer protein | 🗸 | 🗸 | 🗸 | 5 |
| Q8PVI6 | MM1977 | Hypothetical protein | 🗸 | 🗸 | 🗸 | 3 |
| Q8PVG6 | MM1999 | Hypothetical protein | 🗸 | 🗸 | 🗸 | 3 |
| Q8PVG5 | MM2000 | Hypothetical protein | 🗸 | 🗸 | 🗸 | 5 |
| Q8PVD2 | MM2033 | Stomatin-like protein |  |  | 🗸 | 1 |
| Q8PVA2 | MM2069 | Iron III dicitrate binding protein | 🗸 | 🗸 | 🗸 | 1 |
| Q8PV50 | MM2124 | Rpl3p | 🗸 | 🗸 |  | 3 |
| Q8PUU5 | MM2234 | Hypothetical protein | 🗸 | 🗸 | 🗸 | 1 |
| Q8PUM8 | MM2305 | Na/proline symporter | 🗸 | 🗸 | 🗸 | 0 |
| Q8PUL4 | MM2320 | EchA |  |  | 🗸 | 1 |
| Q8PUL3 | MM2321 | EchB |  | 🗸 | 🗸 | 2 |
| Q8PU81 | MM2460 | Dipeptide oligopeptide binding | 🗸 | 🗸 | 🗸 | 2 |
| F1SVH9 | MM2481 | FpoM, F420H2 dehydrogenase |  | 🗸 | 🗸 | 2 |
| F1SVK0 | MM2482 | FpoL, F420H2 dehydrogenase |  | 🗸 | 🗸 | 0 |
| F1SVE0 | MM2483 | FpoK, F420H2 dehydrogenase | 🗸 |  | 🗸 | 0 |
| Q8PU59 | MM2487 | FpoH, F420H2 dehydrogenase |  |  | 🗸 | 1 |
| Q8PTZ0 | MM2567 | ABC transporter | 🗸 | 🗸 | 🗸 | 6 |
| Q8PTK5 | MM2710 | Hypothetical protein | 🗸 | 🗸 | 🗸 | 5 |
| Q8PTI7 | MM2728 | Hypothetical protein |  |  | 🗸 | 5 |
| Q8PT29 | MM2889 | Hypothetical protein |  |  | 🗸 | 0 |
| Q8PT25 | MM2893 | Hypothetical protein | 🗸 | 🗸 | 🗸 | 4 |
| Q8PSQ2 | MM3024 | Hypothetical protein | 🗸 | 🗸 | 🗸 | 33 |

As described in Materials and Methods section, SignalP 3.0 algorithm (Bendtsen et al., 2004b) was employed to predict which of the proteins observed (see Tables 1, 2, S-1, and S-2) are predicted to be secreted. Those proteins were submitted to the NetNGlyc 1.0 server (Blom et al., 2004) to predict potential *N-*glycosylation.
